# Dissipative Majorana Quantum Wires

**DOI:** 10.1016/j.isci.2019.10.025

**Published:** 2019-10-17

**Authors:** Yizhen Huang, Alejandro M. Lobos, Zi Cai

**Affiliations:** 1 Wilczek Quantum Center and Key Laboratory of Artificial Structures and Quantum Control, School of Physics and Astronomy, Shanghai Jiao Tong University, Shanghai 200240, China; 2 Facultad de Ciencias Exactas y Naturales, Universidad Nacional de Cuyo and CONICET, Mendoza 5500, Argentina

**Keywords:** State of Matter, Quantum Phenomena, Quantum Mechanics

## Abstract

In this paper, we formulate and quantitatively examine the effect of dissipation on topological systems. We use a specific model of Kitaev quantum wire with an onsite Ohmic dissipation and perform a numerically exact method to investigate the effect of dissipation on the topological features of the system (e.g., the Majorana edge mode) at zero temperature. We find that even though the topological phase is robust against weak dissipation as it is supposed to be, it will eventually be destroyed by sufficiently strong dissipation via either a continuous quantum phase transition or a crossover depending on the symmetry of the system. The dissipation-driven quantum criticality has also been discussed.

## Highlights

• Dissipation can destroy topological phase either by phase transitions or by crossovers

• The phase transitions or crossovers are determined by the symmetry of system

## Graphical Abstract

**Figure:**
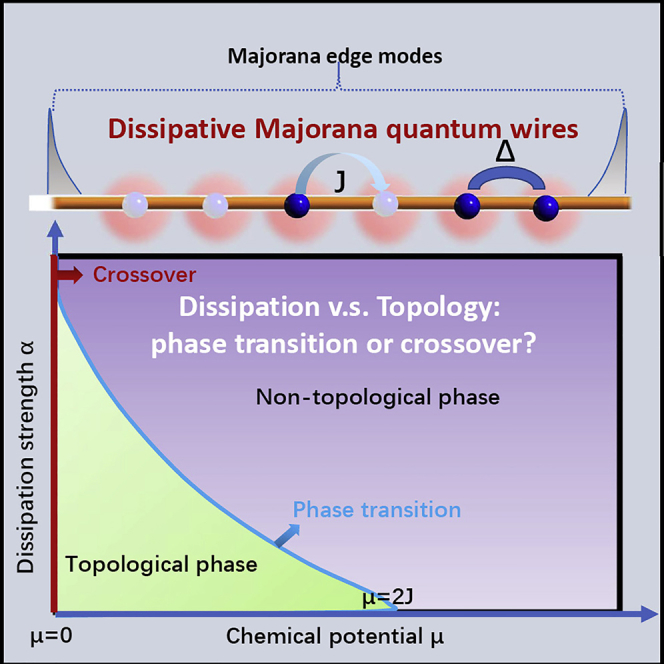

## Introduction

Topological quantum phases of matter are among the most notable phenomena in condensed matter physics (Thouless, 1998). Instead of being classified by symmetries and their spontaneous breaking, topological phases of matters are identified by nonlocal topological orders that are immune to local perturbations (Wen, 2004). The intrinsic stability of the topological features in the underlying systems makes them a promising platform for quantum computation and information processing (Nayak et al., 2008). One of the major obstacles for the realization of a practical quantum computer is that quantum systems are inevitably coupled to their surroundings, which gives rise to dissipation and decoherence that is detrimental to the quantum coherence (Schlosshauer, 2007). Since coupling to the environment tends to drive a quantum system to be classical, although topological phases are quantum in nature, it is natural to expect that sufficiently large bath-induced dissipation and decoherence will eventually destroy the topological phases in spite of their robustness against small perturbations. The question is: How large? And does the system experience a crossover or a phase transition during this process?

Understanding a topological system immersed in an environment is not only of fundamental interest of topology physics itself, but also of immense practical significance in quantum simulation and information processing and hence deserves quantitative studies rather than qualitative arguments. However, quantitatively examining the problem poses multiple challenges: an open quantum system coupled to an environment is, in general, a genuine interacting system even if the system Hamiltonian itself is noninteracting: the bath will inevitably induce effective interactions between the system particles. Generalization of topological phases to noninteracting open systems is a non-trivial problem (Bardyn et al., 2018, Budich and Diehl, 2015, Huang and Arovas, 2014, Uhlmann, 1986, Viyuela et al., 2014a, Viyuela et al., 2014b), let alone the interacting cases (Grusdt, 2017, Trebst et al., 2007). As a consequence, most theoretical efforts are based on various approximations (e.g., the Born-Markovian and weak system-bath [SB] coupling approximations [Bardyn et al., 2012, Budich et al., 2015, Diehl et al., 2011, Linzner et al., 2016]) or simplifications (e.g., treating the bath as classical noises [Goldstein and Chamon, 2011, Hu et al., 2015, Knapp et al., 2016, Liu et al., 2017, Pedrocchi and DiVincenzo, 2015, Rainis and Loss, 2012]).

In this paper, we investigate the fate of a topological phase in the presence of dissipation by performing a numerically exact method, which allows us to investigate this complex interacting quantum open system in both Markovian and non-Markovian cases ranging from weak to strong SB coupling regimes in a unified picture. We choose our system Hamiltonian as a one-dimensional (1D) Kitaev model (Kitaev, 2001), a prototypical example to illustrate nontrivial topology and edge state, whereas the environment is modeled by sets of harmonic oscillators following Caldeira-Leggett's seminal work (Caldeira and Leggett, 1981, Caldeira and Leggett, 1983a, Caldeira and Leggett, 1983b, Leggett et al., 1987). The key outcome of this paper is that, by increasing the dissipation strength, the topological phase can be destroyed via either a continuous quantum phase transition or a crossover depending on the symmetries of our model. The fate of Majorana fermions in the presence of dissipation has also been investigated. In addition, using the framework of Abelian bosonization, we provide an analytical description of the interplay between pairing, dissipation, and interaction in our model.

## Results

The Hamiltonian of a dissipative system contains three parts and is expressed as *H*_*tot*_ = *H*_*s*_ + *H*_*b*_ + *H*_*sb*_. *H*_*s*_ is the system Hamiltonian chosen as a Kitaev wire and is given as follows:

(Equation 1) $$H_{s}=\sum_{\langle ij\rangle }\left\{-J\left(c_{i}^{†}c_{j}+c_{j}^{†}c_{i}\right)-\text{Δ}\left(c_{i}^{†}c_{j}^{†}+c_{j}c_{i}\right)\right\}-\mu \sum_{i}n_{i},$$

where *c*_*i*_ ($c_{i}^{†}$) are the annihilation (creation) operators of spinless fermions at site *i*, *J* (Δ) denotes the hopping (pairing) amplitude between nearest neighboring sites, and μ is the chemical potential. In the following, we choose Δ = *J* for simplicity. On each site *i*, a fermion additionally couples to a local bath (modeled by a set of harmonic oscillators) via its density operator *n*_*i*_. The Hamiltonians describing each local bath and system-bath coupling read as follows:

(Equation 2) $$H_{b}=\sum_{i,k}\frac{P_{ik}^{2}}{2m_{k}}+\frac{1}{2}m_{k}\omega_{k}^{2}X_{ik}^{2},$$

(Equation 3) $$H_{sb}=\sum_{i,k}\left[\frac{\lambda_{k}}{\sqrt{2m_{k}\omega_{k}}}\left(n_{i}-\frac{1}{2}\right)X_{ik}\right],$$

where *X*_*ik*_ (*P*_*ik*_) denotes the coordinate (momentum) operator of the bath harmonic oscillator with modes *ω*_*k*_ on site *i*. The baths around different system sites are independent of each other but are characterized by the same Ohmic spectral function: $J\left(\omega \right)=\pi \sum_{k}\frac{\lambda_{k}^{2}}{2m_{k}\omega_{k}}\delta \left(\omega -\omega_{k}\right)=\pi \alpha \omega$ for 0 < *ω* < *ω*_*D*_ and *J*(*ω*) = 0 otherwise. *ω*_*D*_ is a hard frequency cutoff chosen as *ω*_*D*_ = 10*J*, and α is the dissipation strength.

Integrating out the bath degrees of freedom leads to a retarded interaction term in imaginary time. The total system (system + bath) is assumed to be in thermal equilibrium at temperature *T* = 1/*β*; thus, the partition function of the total system takes the form $Z=\text{Tr}e^{-\beta H_{tot}}=Z_{B}\times {\text{Tr}}_{s}\rho_{s}$, where *Z*_*B*_ is the partition function for the free bosons of the bath and *ρ*_*s*_ is the reduced density matrix of the system (Hänggi and Ingold, 2006, Hänggi et al., 2008) and takes the form given below:

(Equation 4) $$\rho_{s}=e^{-\beta H_{s}+\int_{0}^{\beta }d\tau \int_{0}^{\beta }d\tau^{\prime }\sum_{i}\left(n_{i}\left(\tau \right)-\frac{1}{2}\right)D\left(\tau -\tau^{\prime }\right)\left(n_{i}\left(\tau^{\prime }\right)-\frac{1}{2}\right)}.$$

The effect of dissipation is encapsulated in the onsite retarded interaction in Equation (4) characterized by the site-independent kernel function of the Ohmic spectrum (Winter et al., 2009) $D\left(\tau \right)=\int_{0}^{\infty }d\omega \frac{J\left(\omega \right)}{\pi }\frac{\text{cosh}\left(\frac{\omega \beta }{2}-\omega \left|\tau \right|\right)}{\text{sinh}\left(\frac{\beta \omega }{2}\right)}$. In the limit of *T* = 0 and $\tau \gg \tau_{c}=2\pi /\omega_{D}$, $D\left(\tau \right)∼1/\tau^{2}$. The reason for the choice of the factor $\frac{1}{2}$ in Equation (3) is that we wish the bath effect to be purely dynamical, such that the equal-time component of the retarded interactions in Equation (4) contribute constants to the system Hamiltonian $\left[{\left(n_{i}-\frac{1}{2}\right)}^{2}=\frac{1}{4}\right]$; thus the bath does not renormalize the Hamiltonian parameters in the system. Experimentally, in the hybrid nanowires, the Ohmic dissipation can be realized via an electrostatic coupling of quantum wire to metallic gates/films (Cazalilla et al., 2006), whereas in the ultracold atomic setup, a three-dimensional Fermi sea can be considered as a microscopic realization of such an Ohmic environment (Malatsetxebarria et al., 2013).

For the dissipationless case (*α* = 0), it is well known that the ground state of Hamiltonian.(1) experiences a QPT from a topologically nontrivial phase to a trivial one at *μ* = 2*J*. In the following, we will focus on the topological non-trivial phase (e.g., *μ* = *J*) and investigate its fate with increasing dissipation using a sign-problem free Quantum Monte Carlo (QMC) simulation with worm update. Since this method applies only to bosonic or spin systems, we first perform the Jordan-Wigner transformation(JWT) to map the Kitaev model into a transverse Ising (TI) model: $H_{s}=-J\;\sum_{i}\sigma_{i}^{x}\sigma_{i+1}^{x}-\frac{\mu }{2}\sum_{i}\sigma_{i}^{z}$ (*σ*_*i*_ the Pauli matrices). This enables us to study this model via QMC simulations with the worm algorithm (Prokof'ev et al., 1998) even in the presence of retarded interaction (see the Supplemental Information for details), which is invariant under JWT (with $n_{i}-\frac{1}{2}$ replaced by $\frac{1}{2}\;\sigma_{i}^{z}$). What we actually simulate is a transverse Ising (TI) model with retarded interaction, and we use its phase diagram to interpret that of the dissipative Kitaev model. Since both the JWT and Gaussian integral are exact, these two models are exactly equivalent and thus share the same phase diagram.

We focus on the ground state (*T* = 0) of the total system. In our QMC simulations, the inverse temperature is scaled as *β* = *L*, corresponding to a dynamical critical exponent *z* = 1, which is indeed the case in the QPT in the dissipationless TI model. The periodic boundary condition (PBC) in our simulations corresponds to PBC/anti-PBC in the Kitaev wire depending on the odd/even parity of the particle number. Our model preserves the parity of the particle number of fermions even in the presence of dissipation, which allows us to restrict our measurement in the even parity subspace, which corresponds to ground state of finite system.

**Figure 1:**
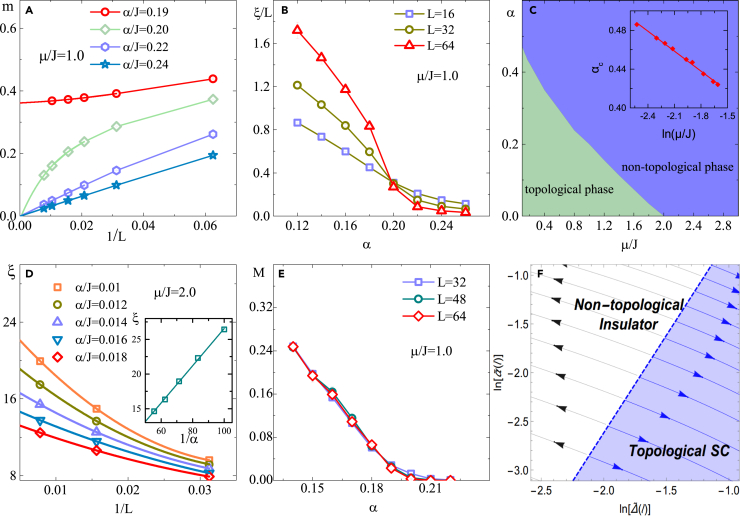
Majorana Quantum Wire in the Presence of Ohmic Dissipation (A) Finite size scaling of the structure factor with different α; (B) correlation length normalized by the size *L* as a function of α; (C) phase diagram of the dissipative Kitaev model (or the equivalent dissipative TI model); the inset shows that for small μ the phase boundary satisfies the relations *α*_*c*_∼*lnμ*, as predicted by the perturbation theory; (D) finite size scaling of the correlation length with different α values near the critical point *μ*_*c*_ = 2*J* of the dissipationless TI model (the inset shows the correlation length as a function of 1/*α* at *μ*_*c*_ = 2*J*); (E) dissipation (α) dependence of the correlation function between the Majorana fermions at the two ends of the chain; (F) RG flow diagram for $\tilde{\text{Δ}}\left(\ell \right)$ and $\tilde{\alpha }\left(\ell \right)$ with an initial *K*_0_ = 0.501; the dashed blue line satisfies *dK*(*ℓ*)/*dℓ* = 0 (e.g., condition $2\pi {\tilde{\text{Δ}}}^{2}=K^{2}\tilde{\alpha }$). *μ* = *J* for (A),(B) and (E), and *β* = *L*.

We first fix the value of *μ* = *J* and increase α. Under the JWT, the topological phase in the Kitaev model can be mapped onto a magnetically ordered phase with spontaneous *Z*_2_ symmetry breaking; therefore, we use the long-range correlation functions $\langle \sigma_{i}^{x}\sigma_{j}^{x}\rangle$ and their Fourier components $S\left(Q\right)=\frac{1}{L^{2}}\sum_{ij}e^{iQ\left(i-j\right)}\langle \sigma_{i}^{x}\sigma_{j}^{x}\rangle$ (structure factor) to identify the QPT induced by dissipation. We define $m=\sqrt{S\left(Q=0\right)}$ as the order parameter of the magnetic ordering phase, which extrapolates to its ground state value *m*_0_ as L=β→∞ in finite size scaling. As shown in Figure 1A, for small α, *m*_0_ is finite, whereas it vanishes in the presence of large dissipation. This dissipation-driven QPT can be further verified by the correlation length ξ, which can be calculated from the structure factors *S*(*Q*) at *Q*_0_ = 0 and *Q*_1_ = 2*π*/*L* (Sandvik, 2010):

(Equation 5) $$\xi =\frac{1}{Q_{1}}\sqrt{\frac{S\left(Q_{0}\right)}{S\left(Q_{1}\right)}-1}$$

The normalized correlation length *ξ*/*L* as a function of α for different system sizes has been plotted in Figure 1B, where we can find a crossing point, indicating a scale-invariant quantum critical point (QCP). As shown in Figure 1C, there are two distinct phases in the phase diagram of this model: a ferromagnetic phase (or topological phase in the fermonic language) and a paramagnetic phase.

It is worthwhile to compare the role of dissipation with that of temperature (T), since both of them tend to suppress quantum fluctuations. Throughout this paper, we focus on the zero temperature properties of the total system (system + bath), where a strong SB coupling might drive the “system” to a mixed state that resembles neither the ground state nor a thermal state with an effective temperature of the “system” Hamiltonian. For an instance, in such a 1D system, the dissipation can drive a continuous phase transition, which is forbidden at any finite temperature. Near the QCP, it is well known that the TI model is a prototype model to illustrate quantum critical matter, whose properties are determined by the QCPs even at a finite temperature (Coleman and Schofield, 2005, Hertz, 1976, Millis, 1993). The question is what happens if the finite T is replaced by dissipation? (Near the QPC, we increase dissipation but fix the temperature of the total system to be zero.) To study this problem, we focus on the QCP of the dissipationless TI model at *μ* = 2*J* and calculate the dependence of the spatial correlation length (ξ) on α in the case of weak dissipation. As shown in the inset of Figure 1D, ξ is proportional to 1/*α* for weak dissipation, similar to the temperature dependence of ξ in quantum critical regime at finite T (Sachdev, 1999). Therefore, the dissipation plays a similar role as temperature near the QCP, whereas a qualitative difference is that, in 1D, the long-range magnetic order is fragile at any finite T but robust against small dissipation.

In the *α*−*μ* phase diagram, the line *μ* = 0 is special as the total Hamiltonian *H*_*tot*_ with *μ* = 0 possesses extra symmetries besides the parity symmetry (e.g., $\left[\hat{S},H_{tot}\right]=0$ with $\hat{S}=\prod_{i}\sigma_{i}^{z}$). At *μ* = 0, at each site *i*, *H*_*tot*_ is invariant under a combined transformation defined as ${\hat{P}}_{i}={\hat{\sigma }}_{i}^{x}{⊗}_{k}{\hat{P}}_{ik}$, where ${\hat{P}}_{ik}$ is the inversion operator for the *k*th mode harmonic oscillator at site i: ${\hat{P}}_{ik}^{-1}X_{ik}{\hat{P}}_{ik}=-X_{ik}$. It is easy to check that each ${\hat{P}}_{i}$ commutes with *H*_*tot*_ ($\left[{\hat{P}}_{i},H_{tot}\right]=0$), indicating infinite number of conserved quantities. Even though both ${\hat{P}}_{i}$ and $\hat{S}$ commute with *H*_*tot*_, they do not commute with each other $\left[\hat{S},{\hat{P}}_{i}\right]\neq 0$, which indicates that all the eigenstates are at least doubly degenerate. In Josephson junction arrays (Douçot et al., 2005, Loffe et al., 2002) and trapped ions (Milman et al., 2007), similar degenerate states with noncommutative conserved quantities have been proposed to be used to construct topologically stable qubits that are robust against decoherence. In our model, these extra symmetries and degeneracies at *μ* = 0 will give rise to remarkable consequences, as we will show in the following.

We focus on the strongly dissipative limit and perform a perturbation analysis of the total Hamiltonian *H*_*tot*_. In the case of *μ* = *J* = 0, different lattice sites are decoupled and for each site, the ground states are doubly degenerate, denoted as “dressed” spin states ($|\tilde{↑}\rangle_{i}$ and $|\tilde{↓}\rangle_{i}$) satisfying the relation $\left|\tilde{↑}\rangle_{i}={\hat{P}}_{i}\right|\tilde{↓}\rangle_{i}$. In the strong dissipative limit $\left\{J,\mu \right\}\ll \left\{\lambda_{k},\omega_{k}\right\}$, one can consider the “system” Hamiltonian *H*_*s*_ as perturbations and derive an effective Hamiltonian $\tilde{H}$ in the 2^*L*^-dimensional constraint Hilbert spaces spanned by the $\left\{{\tilde{\sigma }}_{i}^{z}\right\}$ eigenbasis of the “dressed” spin (see the Supplemental Information for details). In the first-order perturbation, the effective Hamiltonian can be written in terms of the Pauli operators of the “dressed” spin ${\tilde{\sigma }}_{i}$ as $\tilde{H}=\sum_{i}\left[-\tilde{J}{\tilde{\sigma }}_{i}^{x}{\tilde{\sigma }}_{i+1}^{x}-\frac{\tilde{\mu }}{2}{\tilde{\sigma }}_{i}^{z}\right]$, where the effective coupling is strongly suppressed by dissipation $\tilde{J}={\left(\frac{a}{\text{Ω}}\right)}^{\alpha }J$ with Ω and *a* the UV and infrared frequency cutoff of the bath (see Supplemental Information), whereas the chemical potential is not $\tilde{\mu }=\mu$. This perturbative result indicates that, at strongly dissipative limit, the phase boundary occurs at $\alpha_{c}∼-\ln\;\mu$, which agrees with our numerical results. Another prediction is the absence of quantum phase transition at *μ* = 0, indicating that, at this point, dissipation cannot completely destroy the topological phase at zero temperature. This robustness is related to the special symmetries and infinite conserved quantities even in the presence of dissipation, as we analyzed earlier.

Up to now, our discussion was based on spin models. Even though the long-range magnetic correlations can be considered as an indicator of the topological phase in the fermionic counterpart under JWT, they are not directly physically observable in the Kitaev model since they involve nonlocal correlations of string operators in terms of fermion operators. In general, a topological phase is characterized by distinct integer values of topological invariant quantities. However, for an interacting open quantum system as in our case, it is challenging to define or calculate such a topological invariant quantity. An alternative feature of a topological phase is the existence of robust zero modes localized at the edges, known as Majorana edge mode in the Kitaev model. The existence of Majorana mode is characterized by the nonvanishing correlations between the Majorana fermions defined at two ends of the 1D lattice with open boundary condition: $M=-i\langle \gamma_{1}\gamma_{2L}\rangle$ with $\gamma_{2i-1}=c_{i}+c_{i}^{†}$ and $\gamma_{2i}=i\left(c_{i}^{†}-c_{i}\right)$, the Majorana fermion operators.

Understanding the effect of the environment on Majorana fermions is crucial and of practical significance for current experiments in solid-state devices (Churchill et al., 2013, Deng et al., 2012, Mourik et al., 2012, Nadj-Perge et al., 2014, Sun et al., 2016). A variety of theoretical methods have been employed to study this problem under various approximations (Goldstein and Chamon, 2011, Hu et al., 2015, Knapp et al., 2016, Liu et al., 2017, Pedrocchi and DiVincenzo, 2015, Rainis and Loss, 2012), most of which focus on the dynamical aspect of the environment, modeled by classical noise that heat the system and destroys the topology via a crossover. Here, we focus on the other aspect, dissipation, of environment, which is relevant for the low-temperature steady-state properties. Recently, the effect of dissipation on the tunneling of Majorana fermion has been discussed analytically (Matthews et al., 2014). Here, we calculate the quantity $\langle \gamma_{1}\gamma_{2L}\rangle$ and use it to characterize the topological phase and Majorana edge mode in our dissipative Kitaev model. In our QMC simulations this quantity can be expressed in terms of the spin operators $M=-i\langle \gamma_{1}\gamma_{2L}\rangle =\langle \sigma_{1}^{x}\sigma_{L}^{x}\prod_{i=1}^{L}\sigma_{i}^{z}\rangle$. The QMC measurement is restricted to the even parity subspace. M as a function of α for different system sizes is shown in Figure 1E, which reveals that M vanishes at a critical *α*_*c*_, whose value agrees with the QCP identified by the correlation lengths. In summary, the fate of Majorana edge modes in the presence of dissipation indicates that it will drive a topological nontrivial phase into a trivial one without Majorana edge mode via a continuous QPT.

To get a better understanding of the dissipation-driven QPT, we perform the bosonization technique in which the effective field theory of the system Hamiltonian can be expressed in terms of two bosonic fields *ϕ*(*x*) and *θ*(*x*) (Lobos et al., 2012): $H_{s}=\int dx\left\{\frac{v}{2\pi }\left[\frac{1}{K}{\left(\partial_{x}\phi \right)}^{2}+K{\left(\partial_{x}\theta \right)}^{2}\right]+\tilde{\text{Δ}}\rho_{0}\text{sin}\left(2\theta \right)\right\}$, with the Luttinger parameter *K*, the sound velocity *v*, and a dimensionless parameter $\tilde{\text{Δ}}$ characterizing the strength of the p-wave pairing. If the average particle number is away from 1/2(*μ*≠0), we can ignore the spatially fast oscillating terms. The effective action describing the retarded interaction induced by dissipation can also be expressed in the bosonization language (Cazalilla et al., 2006): $S_{\text{ret}}=-\frac{\tilde{\alpha }}{a_{0}}\int dx\int d\tau d\tau^{\prime }\frac{\cos\;2\left[\phi \left(x,\tau \right)-\phi \left(x,\tau^{\prime }\right)\right]}{{\left(\tau -\tau^{\prime }\right)}^{2}}$ where the dimensionless parameter $\tilde{\alpha }$ is proportional to the dissipation strength. Finally, we obtain the effective action of the dissipative Kitaev model $S_{\text{eff}}=\int_{0}^{\beta }d\tau \left[\int dx\frac{1}{i\pi }\dot{\theta }\partial_{x}\phi +H_{s}\left(\tau \right)\right]+S_{\text{ret}}$.

To study the interplay between the dissipation and p-wave pairing, we perform the standard perturbative renormalization group (RG) procedure to analyze the RG-flow of the parameters $\tilde{\alpha }$, $\tilde{\text{Δ}}$, *v*, and *K*, and their flow equations read (see the Supplemental Information):

(Equation 6) $$\begin{array}{l} \frac{d\tilde{\text{Δ}}\left(\ell \right)}{d\ell }=\left[2-\frac{1}{K\left(\ell \right)}\right]\tilde{\text{Δ}}\left(\ell \right),\\ \frac{d\tilde{\alpha }\left(\ell \right)}{d\ell }=\left[1-2K\left(\ell \right)\right]\tilde{\alpha }\left(\ell \right)\\ \frac{dv\left(\ell \right)}{d\ell }=-2\pi K\left(\ell \right)v\left(\ell \right)\tilde{\alpha }\left(\ell \right),\\ \frac{dK\left(\ell \right)}{d\ell }=4\pi^{2}{\tilde{\text{Δ}}}^{2}\left(\ell \right)-2\pi K^{2}\left(\ell \right)\tilde{\alpha }\left(\ell \right). \end{array}$$

The main results of our model can be illustrated by the flow equations Equation (6), from which we can find a phase transition point at *K*_*c*_ = 1/2. For *K*(*ℓ*) > *K*_*c*_, $\tilde{\text{Δ}}\left(\ell \right)$ flows to the strong coupling limit while $\tilde{\alpha }\left(\ell \right)$ goes to zero. $\tilde{\text{Δ}}\left(\ell \right)$ couples to the sin2*θ* terms in the Hamiltonian; once it becomes relevant, the field θ becomes pinned to one of the two degenerate energy minima of the potential: *θ* = −*π*/4 or 3*π*/4, indicating a spontaneous *Z*_2_ symmetry breaking observed in our QMC simulations for small α. For *K*(*ℓ*) < *K*_*c*_, the dissipation is relevant while the effect of the pairing is suppressed in the RG sense; therefore, this phase can be understood as a dissipative Luttinger liquid, which has been investigated analytically (Castro Neto et al., 1997, Cazalilla et al., 2006, Malatsetxebarria et al., 2013) and numerically (Cai et al., 2014). The intertwined effects between the dissipation and pairing can be found from the RG flow equations of *K* and *v*, from which we can find that the velocity is only renormalized by dissipation, since it essentially breaks the Lorentz invariance of the Luttinger Liquid term. Dissipation makes the plasmon velocity become slower; a similar effect has been discussed in the Coulomb drag (Cazalilla et al., 2006, Lobos and Giamarchi, 2011). By solving the RG flow equations, we plot the RG flow diagram as shown in Figure 1F, which shows the diverging RG flows of the dissipation and pairing parameters in the different regions of the phase space.

## Discussion

Our results may be relevant with current experiments of topological superfluid and Majorana fermions in both solid state and ultracold atomic setups. In most cases of solid state experiments, the strength of dissipation is difficult to be controlled and tuned, so as an experimental realization of the dissipative Kitaev model, we follow the implementation of a topological superfluid proposed by Nascimbene (Nascimbene, 2013) and estimate the relevant parameters in corresponding ultracold atomic setups. As proposed by Nascimbene (2013), the 1D Kitaev model can be realized by loading 1D gas of fermionic atoms (e.g., ^161^Dy) into a spin-dependent optical superlattice immersed in an environment composed of a two-dimensional (2D) condensate of Feshbach molecules. In an optical lattice with wavelength *λ* = 530 nm and the lattice depth along x-direction *V*_*x*_ = 5*E*_*r*_ ($E_{r}=\frac{h^{2}}{2m\lambda^{2}}=4.38\left(2\pi \right)$ kHz is the recoil energy in this setup), by tuning the scattering length of the fermions and the density of the 2D molecules, one can realize the Kitaev model with parameters $J∼\text{Δ}\approx 0.1E_{r}$, which corresponds to an energy of *k*_*B*_×21nK. The 2D condensate of Feshbach molecules induces the attractive interactions between the 1D fermions; the static or momentum-dependent part of this bath-induced interaction gives rise to a p-wave pairing, whereas the dynamical or frequency-dependent part plays a role of quantum dissipation. It has been shown that the quantum environment composed of Bogoliubov quasiparticles in a 2D condensate can give rise to Ohmic dissipation (Dalla Torre et al., 2010). By tuning the scattering length between the fermions and molecules, one can realize a dissipation strength α comparable with Δ and *J*. One of the major challenges in the experimental implementation is the finite temperature effect: to observe the dissipation-induced phase transition, the temperature needs to be lower than 20 nK, still below the current experimental limit of the cold fermionic systems.

### Conclusion and Outlook

In summary, we have studied the effect of dissipation on topological quantum phases by considering a specific model of Kitaev quantum wire with onsite Ohmic dissipation and found that the topological phase in this model will eventually be destroyed via either a continuous QPT or a crossover depending on the symmetry of the system. Some avenues for further investigations can be suggested. The first and most important question is the generality of the above-mentioned results, whether it applies to other topological models with different kind of dissipation. An important feature of our model is that a system particle interacts with the bath via its density operators; this dissipation process preserves the total number (also the parity) of the particles in the system. We expect that our results hold for this type of symmetry-protected dissipation, whereas for other dissipation mechanisms (e.g., the particle loss) that break these symmetries, the conclusion may be different. This point needs to be verified numerically, which requires new methods and models (Yan et al., 2018). Another important ingredient still missing is a proper definition of a topological invariant (an integer number) for these interacting open quantum systems, which may provide more direct evidence of the topological phases and topological QPT compared with the existence of edge modes. This topological number needs to be not only well defined but also computable in our practical numerical simulations. A real-time dynamics of the model is also an interesting question, which is closely related to the decoherence problem in topological quantum computation and has been explored recently (Weisbrich et al., 2019). Last but not the least, our work also raises an interesting question whether non-trivial topological properties could exist only in a subsystem of reduced dimensionality spatially embedded in a larger non-topological system with an inhomogeneous Hamiltonian, and if so, how to identify this subsystem topological phases and what distinguishes them from the conventional topological matters.

## Methods

All methods can be found in the accompanying Transparent Methods supplemental file.

## Supplemental Information

Document S1. Transparent Methods

## References

[bib1] Bardyn C.-E., Baranov M.A., Rico E., İmamoğlu A., Zoller P., Diehl S. (2012). Majorana modes in driven-dissipative atomic superfluids with a zero Chern number. Phys. Rev. Lett..

[bib2] Bardyn C.-E., Wawer L., Altland A., Fleischhauer M., Diehl S. (2018). Probing the topology of density matrices. Phys. Rev. X.

[bib3] Budich J.C., Diehl S. (2015). Topology of density matrices. Phys. Rev. B.

[bib4] Budich J.C., Zoller P., Diehl S. (2015). Dissipative preparation of Chern insulators. Phys. Rev. A.

[bib5] Cai Z., Schollwöck U., Pollet L. (2014). Identifying a bath-induced bose liquid in interacting spin-boson models. Phys. Rev. Lett..

[bib6] Caldeira A.O., Leggett A.J. (1981). Influence of dissipation on quantum tunneling in macroscopic systems. Phys. Rev. Lett..

[bib7] Caldeira A.O., Leggett A.J. (1983). Quantum tunnelling in a dissipative system. Ann. Phys..

[bib8] Caldeira A.O., Leggett A.J. (1983). Path integral approach to quantum Brownian motion. Phys. A.

[bib9] Castro Neto A.H., de Chamon C., Nayak C. (1997). Open Luttinger liquids. Phys. Rev. Lett..

[bib10] Cazalilla M.A., Sols F., Guinea F. (2006). Dissipation-driven quantum phase transitions in a Tomonaga-Luttinger liquid electrostatically coupled to a metallic gate. Phys. Rev. Lett..

[bib11] Churchill H.O.H., Fatemi V., Grove-Rasmussen K., Deng M.T., Caroff P., Xu H.Q., Marcus C.M. (2013). Superconductor-nanowire devices from tunneling to the multichannel regime: zero-bias oscillations and magnetoconductance crossover. Phys. Rev. B.

[bib12] Coleman P., Schofield A.J. (2005). Quantum criticality. Nature.

[bib13] Deng M.T., Yu C.L., Huang G.Y., Larsson M., Caroff P., Xu H.Q. (2012). Anomalous zero-bias conductance peak in a NbInSb nanowireNb hybrid device. Nano Lett..

[bib14] Diehl S., Rico E., Baranov M.A., Zoller P. (2011). Topology by dissipation in atomic quantum wires. Nat. Phys..

[bib15] Douçot B., Feigel’man M.V., Ioffe L.B., Ioselevich A.S. (2005). Protected qubits and Chern-Simons theories in Josephson junction arrays. Phys. Rev. B.

[bib16] Goldstein G., Chamon C. (2011). Decay rates for topological memories encoded with Majorana fermions. Phys. Rev. B.

[bib17] Grusdt F. (2017). Topological order of mixed states in correlated quantum many-body systems. Phys. Rev. B.

[bib18] Hänggi P., Ingold G.-L. (2006). Quantum Brownian motion and the third law of thermodynamics. Acta Phys. Pol. B.

[bib19] Hänggi P., Ingold G.-L., Talkner P. (2008). Finite quantum dissipation: the challenge of obtaining specific heat. New J. Phys..

[bib20] Hertz J.A. (1976). Quantum critical phenomena. Phys. Rev. B.

[bib21] Hu Y., Cai Z., Baranov M.A., Zoller P. (2015). Majorana fermions in noisy Kitaev wires. Phys. Rev. B.

[bib22] Huang Z., Arovas D.P. (2014). Topological indices for open and thermal systems via Uhlmann’s phase. Phys. Rev. Lett..

[bib23] Kitaev A.Y. (2001). ,Unpaired Majorana fermions in quantum wires. Phys.-Uspekhi.

[bib24] Knapp C., Zaletel M., Liu D.E., Cheng M., Bonderson P., Nayak C. (2016). The nature and correction of diabatic errors in anyon braiding. Phys. Rev. X.

[bib25] Leggett A.J., Chakravarty S., Dorsey A.T., Fisher M.P.A., Garg A., Zwerger W. (1987). Dynamics of the dissipative two-state system. Rev. Mod. Phys..

[bib26] Linzner D., Wawer L., Grusdt F., Fleischhauer M. (2016). Reservoir-induced Thouless pumping and symmetry-protected topological order in open quantum chains. Phys. Rev. B.

[bib27] Liu C.-X., Sau J.D., Das Sarma S. (2017). Conductance feature smearing and anisotropic suppression of induced superconductivity in a Majorana nanowire. Phys. Rev. B.

[bib28] Lobos A.M., Giamarchi T. (2011). Superconductor-to-insulator transition in linear arrays of Josephson junctions capacitively coupled to metallic films. Phys. Rev. B.

[bib29] Lobos A.M., Lutchyn R.M., Das Sarma S. (2012). Interplay of disorder and interaction in Majorana quantum wires. Phys. Rev. Lett..

[bib30] Loffe L., Felgelman M., Loselevich A., Ivanov D., Troyer M., Blatter G. (2002). Topologically protected quantum bits using Josephson junction arrays. Nature.

[bib31] Malatsetxebarria E., Cai Z., Schollwöck U., Cazalilla M.A. (2013). Dissipative effects on the superfluid-to-insulator transition in mixed-dimensional optical lattices. Phys. Rev. A.

[bib32] Matthews P., Ribeiro P., García-García A.M. (2014). Dissipation in a simple model of a topological Josephson junction. Phys. Rev. Lett..

[bib33] Millis A.J. (1993). Effect of a nonzero temperature on quantum critical points in itinerant fermion systems. Phys. Rev. B.

[bib34] Milman P., Maineult W., Guibal S., Guidoni L., Douçot B., Ioffe L., Coudreau T. (2007). Topologically decoherence-protected qubits with trapped ions. Phys. Rev. Lett..

[bib35] Mourik V., Zuo K., Frolov S.M., Plissard S.R., Bakkers E.P.A.M., Kouwenhoven L.P. (2012). Signatures of Majorana fermions in hybrid superconductor-semiconductor nanowire devices. Science.

[bib36] Nadj-Perge S., Drozdov I.K., Li J., Chen H., Jeon S., Seo J., MacDonald A.H., Bernevig B.A., Yazdani A. (2014). .Observation of Majorana fermions in ferromagnetic atomic chains on a superconductor. Science.

[bib37] Nascimbene S. (2013). Realizing one-dimensional topological superfluids with ultracold atomic gases. J. Phys. B.

[bib38] Nayak C., Simon S.H., Stern A., Freedman M., Das Sarma S. (2008). Non-Abelian anyons and topological quantum computation. Rev. Mod. Phys..

[bib39] Pedrocchi F.L., DiVincenzo D.P. (2015). Majorana braiding with thermal noise. Phys. Rev. Lett..

[bib40] Prokof’ev N.V., Svistunov B.V., Tupitsyn I.S. (1998). Worm algorithm in quantum Monte Carlo simulations. Phys. Lett. A.

[bib41] Rainis D., Loss D. (2012). Majorana qubit decoherence by quasiparticle poisonings. Phys. Rev. B.

[bib42] Sachdev S. (1999). Quantum Phase Transitions.

[bib43] Sandvik A.W. (2010). Computational studies of quantum spin systems. AIP Conf. Proc..

[bib44] Schlosshauer M. (2007). Decoherence.

[bib45] Sun H.-H., Zhang K.-W., Hu L.-H., Li C., Wang G.-Y., Ma H.-Y., Xu Z.-A., Gao C.-L., Guan D.-D., Li Y.-Y. (2016). Majorana zero mode detected with spin selective Andreev reflection in the vortex of a topological superconductor. Phys. Rev. Lett..

[bib46] Thouless D. (1998). Topological Quantum Numbers in Nonrelativistic Physics.

[bib47] Dalla Torre E.G., Demler E., Giamarchi T., Altman E. (2010). Quantum critical states and phase transitions in the presence of non-equilibrium noise. Nat. Phys..

[bib48] Trebst S., Werner P., Troyer M., Shtengel K., Nayak C. (2007). Breakdown of a topological phase: quantum phase transition in a loop gas model with tension. Phys. Rev. Lett..

[bib49] Uhlmann A. (1986). Parallel transport and quantum holonomy along density operators. Rep. Math. Phys..

[bib50] Viyuela O., Rivas A., Martin-Delgado M.A. (2014). Two-dimensional density-matrix topological fermionic phases: topological Uhlmann numbers. Phys. Rev. Lett..

[bib51] Viyuela O., Rivas A., Martin-Delgado M.A. (2014). Uhlmann phase as a topological measure for one-dimensional fermion systems. Phys. Rev. Lett..

[bib52] Weisbrich H., Belzig W., Rastelli G. (2019). Decoherence and relaxation of topological states in extended quantum Ising models. SciPost Phys..

[bib53] Wen X. (2004). Quantum Field Theory of Many-Body Systems: From the Origin of Sound and Light.

[bib54] Winter A., Rieger H., Vojta M., Bulla R. (2009). Quantum phase transition in the Sub-Ohmic Spin-Boson Model: quantum Monte Carlo Study with a continuous imaginary time cluster algorithm. Phys. Rev. Lett..

[bib55] Yan Z., Pollet L., Lou J., Wang X., Chen Y., Cai Z. (2018). Interacting lattice systems with quantum dissipation: a quantum Monte Carlo study. Phys. Rev. B.

