# Supplementary material for: Dissipative Majorana Quantum Wires
Source: iScience. 2019 Oct 17;21:241–8. doi: 10.1016/j.isci.2019.10.025 (PMC6838469; doi:10.1016/j.isci.2019.10.025)
Supplement: Document S1. Transparent Methods [file mmc1.pdf]

ISCI, Volume *21*

Supplemental Information

Dissipative Majorana Quantum Wires

Yizhen Huang, Alejandro M. Lobos, and Zi Cai

Supplemental Material for "Dissipative Majorana quantum wires"

Transparent method

I. QMC METHOD FOR DISSIPATIVE QUANTUM SYSTEMS

We perform the QMC algorithm to study the transverse Ising model with dissipation-induced retarded interaction. Compared to the standard worm QMC algorithm, the only difference here is the calculation of the integrals resulting from the retardation and multiplying these positive factors into the QMC acceptance ratio during the updates of the samplings. As a consequence, as long as the system Hamiltonian is positive definite in the QMC sampling (for instance, the TI model in our case), the corresponding model with retarded interaction is also free from sign problem, where the QMC is known as an unbiased numerical method giving rise to reliable results.

Dissipationless case: We first focus on the case without dissipation. The Hamiltonian $\hat{H}$ can be decomposed as $\hat{H} = -\hat{T} + \hat{V}$, with $\hat{T}$ the off-diagonal terms and V is the diagonal ones under the basis of $\hat{\sigma}_z$ eigenstates $|\tilde{\sigma}\rangle = |\sigma_1\sigma_2\cdots\sigma_L\rangle$. In the interacting picture, the partition function $Z = \mathbf{Tr}e^{-\beta\hat{H}}$ can be expanded as

$$Z = \mathbf{Tr}\sum_{n=0}^{\infty}\int_0^{\beta}d\tau_n\int_0^{\tau_n}d\tau_{n-1}\cdots\int_0^{\tau_2}d\tau_1 e^{-\tau_1\hat{V}}\hat{T}e^{-(\tau_2-\tau_1)\hat{V}}\cdots e^{-(\tau_n-\tau_{n-1})\hat{V}}\hat{T}e^{-(\beta-\tau_n)\hat{V}}$$

By inserting a set of complete basis, the partition function can be expressed in terms of the probability functions of space-imaginary time configurations: $Z = \sum_{n=0}^{\infty}\sum_{|\tilde{\sigma}\rangle_1,\cdots|\tilde{\sigma}\rangle_n}\int_0^{\beta}d\tau_n\int_0^{\tau_n}d\tau_{n-1}\cdots\int_0^{\tau_2}d\tau_1 W(\tau_1,\cdots,\tau_n,|\tilde{\sigma}\rangle_1,\cdots,|\tilde{\sigma}\rangle_n)$ where $|\tilde{\sigma}\rangle_m$ denotes the spin configurations between the imaginary time τ_{m-1} and τ_m. For a given configuration: $\{\tau_1,\cdots,\tau_n,|\tilde{\sigma}\rangle_1,\cdots,|\tilde{\sigma}\rangle_n\}$, the corresponding probability

$$W(\tau_1,\cdots,\tau_n,|\tilde{\sigma}\rangle_1,\cdots,|\tilde{\sigma}\rangle_n) = \langle\tilde{\sigma}|_1\hat{T}|\tilde{\sigma}\rangle_2 e^{-(\tau_2-\tau_1)E_{\tilde{\sigma}_2}}\langle\tilde{\sigma}|_2\hat{T}|\tilde{\sigma}\rangle_3\cdots e^{-(\tau_n-\tau_{n-1})E_{\tilde{\sigma}_n}}\langle\tilde{\sigma}_n|\hat{T}|\tilde{\sigma}\rangle_1 e^{-(\beta+\tau_1-\tau_n)E_{\tilde{\sigma}_1}}, \quad (1)$$

where $E_{\tilde{\sigma}_m} = \langle\tilde{\sigma}|_m\hat{V}|\tilde{\sigma}\rangle_m$ is the diagonal energy for spin configuration between τ_m and τ_{m-1}. As long as $\hat{T}$ is a positive definite operator (as the transverse Ising model in our case), $W(\tau_1,\cdots,\tau_n,|\tilde{\sigma}\rangle_1,\cdots,|\tilde{\sigma}\rangle_n)$ is always positive. As a consequence, we can perform the importance sampling to evaluate the average value of physical quantities over a few portion instead of full configuration space .

With dissipation: We assume that the total system (system+environment), is in thermodynamic equilibrium and its partition function can be expressed as: $Z = \mathtt{Tr}_{\{\tilde{\sigma}\},\{a_{ik}\}}e^{-\beta\hat{H}_{\rm tot}}$, where $\{\tilde{\sigma}\} = \{\tau_1,\cdots,\tau_n,|\tilde{\sigma}\rangle_1,\cdots,|\tilde{\sigma}\rangle_n\}$ represent a imaginary time-space configuration.

As shown in the main text, integrating out the bath degrees of freedom introduces a retarded interaction in imaginary time, which is diagonal in the σ_z−basis. As a consequence, it does not require a Taylor expansion, but only contributes a factor for any given spin configuration: $S_{ret}(\{\tilde{\sigma}\}) = \int_0^{\beta}d\tau\int_0^{\beta}d\tau'\sum_i\sigma_i(\tau')D(\tau-\tau')\sigma_i(\tau)$ and the partition function in the presence of retarded interaction reads

$$Z = Z_B\sum_{\{\tilde{\sigma}\}}W(\{\tilde{\sigma}\})e^{-S_{\rm ret}(\{\tilde{\sigma}\})}, \quad (2)$$

where the weight $W(\{\tilde{\sigma}\})$ is the same as for the non-dissipative case in Eq.(1). Therefore, as long as the system Hamiltonians is free from sign problem $W(\{\tilde{\sigma}\}) \geq 0$, the corresponding effective action with retarded interaction is also sign problem free (since $e^{-S_{\rm ret}(\{\tilde{\sigma}\})} > 0$).

II. BOSONIZATION METHOD AND RENORMALIZATION GROUP ANALYSIS

b. Bosonization method Following the standard bosonization procedures, the spinless fermion operator can be decomposed as $\psi(x) = e^{-ik_Fx}\psi_L(x) + e^{ik_Fx}\psi_R(x)$, where the right(left)-moving operator $\psi_{L(R)}(x)$ can be expressed in terms bosonic operators $\phi(x)$ and $\theta(x)$ as: $\psi_{R/L}(x) = \frac{U_r}{\sqrt{2\pi a}}e^{\mp i\phi(x)+i\theta(x)}$ where U_r is the Klein factor and a is the short distance cutoff. The density operator of the fermions can be expressed as $\rho(x) = -\frac{1}{\pi}\nabla\phi(x) + \frac{1}{2\pi\alpha}[e^{2i(k_Fx-\phi(x))} + h.c.]$ with $\phi(x)$ and $\theta(x)$ satisfying the relation $[\phi(x), \nabla\theta(x')] = i\pi\delta(x-x')$.

Here, we provide technical details on the bosonization method applied to the quantum nanowire with Ohmic dissipation, and give details on the derivation of the 2-loop RG flow equations. The methods used in this Supplemental Material are standard bosonization and RG techniques that are explained in textbooks[? ?]

We focus on the effect of the superconducting pairing $H_{\rm p}$ and dissipation terms as perturbations to the fixed-point Eucildean action S_0. We start by writing the total partition function of the system

$$Z = \int \mathcal{D}\left[\theta,\phi\right]\, e^{-S_0 - S_{\rm p} - S_{\rm ret}}, \tag{3}$$

where S_0 is the Euclidean action corresponding to the fixed-point Hamiltonian H_0

$$S_0 = \int_0^L dx \int_0^\beta d\tau \left[\frac{1}{i\pi}\partial_\tau\theta\partial_x\phi + \frac{v}{2\pi K}\left(\partial_x\phi\right)^2 + \frac{vK}{2\pi}\left(\partial_x\theta\right)^2\right], \tag{4}$$

where τ is the imaginary time, and $\beta = 1/T$ is the inverse temperature. The bosonic fields obey the usual commutation relations:

$$\left[\phi\left(x\right),\theta\left(y\right)\right] = -i\frac{\pi}{2}\text{sign}\left(x-y\right). \tag{5}$$

The term $S_{\rm p}$ is the Euclidean action corresponding to the pairing interaction

$$S_{\rm p} = \frac{2v\tilde{\Delta}}{a^2}\int_0^L dx \int_0^\beta d\tau\ \sin 2\theta\left(x,\tau\right),$$

where we have defined the dimensionless coupling $\tilde{\Delta} \equiv \frac{\Delta a}{\pi v}$, and

$$S_{\rm ret} = -\frac{\tilde{\alpha}}{a}\int_0^L dx \int_0^\beta d\tau d\tau'\ \frac{\cos 2\left[\phi\left(x,\tau\right) - \phi\left(x,\tau\right)\right]}{\left(\tau-\tau'\right)^2},$$

is the action of the Ohmic dissipation. In the following, we focus on the limit $L\to\infty$ and $T\to 0$.

For later convenience, we now introduce the scaled fields

$$\phi \to \tilde{\phi} = \frac{\phi}{\sqrt{K}} \tag{6}$$

$$\theta \to \tilde{\theta} = \sqrt{K}\theta \tag{7}$$

which preserve the commutation relation (5), and the compact notation

$$\mathbf{r} = \left(x, v\tau\right)$$

In addition, we define the vertex operators

$$e^{i2\sqrt{K}\tilde{\phi}(\mathbf{r})} = \left(\frac{2\pi a}{L}\right)^K : e^{i2\sqrt{K}\tilde{\phi}(\mathbf{r})} :,$$

$$e^{i2\tilde{\theta}(\mathbf{r})/\sqrt{K}} = \left(\frac{2\pi a}{L}\right)^{1/K} : e^{i2\tilde{\theta}(\mathbf{r})/\sqrt{K}} :,$$

where the notation $:\hat{O}:$ means that the operator $\hat{O}$ is normal-ordered. In terms of the above quantities, the Euclidean

action now writes as

$$S_0 = \int d^2\mathbf{r} \left[-\frac{i}{\pi}\partial_y\tilde{\theta}\partial_x\tilde{\phi} + \frac{1}{2\pi}\left(\partial_x\tilde{\phi}\right)^2 + \frac{1}{2\pi}\left(\partial_x\tilde{\theta}\right)^2 \right], \tag{8}$$

$$S_{\mathrm{p}} = \frac{\tilde{\Delta}}{ia^{2-1/K}} \int d^2\mathbf{r} \left(\frac{2\pi}{L}\right)^{1/K} \left(: e^{i2\tilde{\theta}(\mathbf{r})/\sqrt{K}} : - \mathrm{H.c.} \right), \tag{9}$$

$$S_{\mathrm{ret}} = -\frac{1}{2}\frac{\tilde{\alpha}}{a^{1-2K}} \int_{a<|\mathbf{r}_1-\mathbf{r}_2|} d^2\mathbf{r}_1 d^2\mathbf{r}_2 \; \delta(x_1 - x_2) \left(\frac{2\pi}{L}\right)^{2K} \times \left(\frac{: e^{i2\sqrt{K}\tilde{\phi}(\mathbf{r}_1)} :: e^{-i2\sqrt{K}\tilde{\phi}(\mathbf{r}_2)} :}{(\mathbf{r}_1 - \mathbf{r}_2)^2} + \mathrm{H.c.} \right). \tag{10}$$

We now return to Eq. (3) and expand the partition function in powers of the dimensionless couplings $\tilde{\Delta}$ and $\tilde{\alpha}$

$$Z = Z_0 \times \left\{ 1 + \frac{1}{2!}\left(\frac{\tilde{\Delta}}{ia^{2-1/K}}\right)^2 \int_{a<|\mathbf{r}_1-\mathbf{r}_2|} d^2\mathbf{r}_1 d^2\mathbf{r}_2 \left(\frac{2\pi}{L}\right)^{2/K} \left\langle \left[: e^{i2\tilde{\theta}(\mathbf{r}_1)/\sqrt{K}} : - \mathrm{H.c.} \right] \left[: e^{i2\tilde{\theta}(\mathbf{r}_2)/\sqrt{K}} : - \mathrm{H.c.} \right] \right\rangle_0 , \right.$$
$$\left. + \frac{\tilde{\alpha}}{2a^{1-2K}} \int_{a<|\mathbf{r}_1-\mathbf{r}_2|} d^2\mathbf{r}_1 d^2\mathbf{r}_2 \; \delta(x_1 - x_2) \left(\frac{2\pi}{L}\right)^{2K} \frac{\left\langle : e^{i2\sqrt{K}\tilde{\phi}(\mathbf{r}_1)} :: e^{-i2\sqrt{K}\tilde{\phi}(\mathbf{r}_2)} : \right\rangle_0}{(\mathbf{r}_1 - \mathbf{r}_2)^2} + \mathrm{H.c.} + \ldots \right\} \tag{11}$$

where the averages are taken with respect to the fixed-point action S_0, and where we have used that $\left\langle : e^{i2\tilde{\theta}(\mathbf{r})/\sqrt{K}} : \right\rangle_0 = \left\langle : e^{i2\sqrt{K}\tilde{\phi}(\mathbf{r})} : \right\rangle_0 = 0$.

We now implement the RG transformation by performing an infinitesimal change in the microscopic cutoff a, and asking how the parameters $\left\{K, v, \tilde{\Delta}, \tilde{\alpha}\right\}$ of the model should change in order to preserve the partition function Z. It is convenient to parametrize the RG transformation with a dimensionless continuous variable ℓ, i.e., $a = a(\ell) \equiv a_0 e^{\ell}$. In this way, the parameters of the model become functions of ℓ through their dependence on $a(\ell)$: $\left\{K, v, \tilde{\Delta}, \tilde{\alpha}\right\} \rightarrow \left\{K(\ell), v(\ell), \tilde{\Delta}(\ell), \tilde{\alpha}(\ell)\right\}$. We now focus on the infinitesimal transformation $a(\ell) \rightarrow a(\ell + d\ell) \simeq a(\ell)[1 + d\ell]$, and demand that the equation

$$Z(\ell) = Z(\ell + d\ell), \tag{12}$$

is satisfied [? ?]. To simplify the notation, we denote the integrals over $\mathbf{r}_1$ and $\mathbf{r}_2$ in (11) as

$$I(\ell) = \frac{1}{2}\frac{\left(\tilde{\Delta}(\ell)\right)^2}{(a(\ell))^{4-2/K}} \int_{a(\ell)<|\mathbf{r}_1-\mathbf{r}_2|} d^2\mathbf{r}_1 d^2\mathbf{r}_2 \left(\frac{2\pi}{L}\right)^{2/K} \left[: e^{i2\tilde{\theta}(\mathbf{r}_1)/\sqrt{K}} :: e^{-i2\tilde{\theta}(\mathbf{r}_2)/\sqrt{K}} : + \mathrm{H.c.} \right]$$
$$+ \frac{1}{2}\frac{\tilde{\alpha}(\ell)}{(a(\ell))^{1-2K}} \int_{a(\ell)<|\mathbf{r}_1-\mathbf{r}_2|} d^2\mathbf{r}_1 d^2\mathbf{r}_2 \; \delta(x_1 - x_2) \left(\frac{2\pi}{L}\right)^{2K} \left[\frac{: e^{i2\sqrt{K}\tilde{\phi}(\mathbf{r}_1)} :: e^{-i2\sqrt{K}\tilde{\phi}(\mathbf{r}_2)} :}{(\mathbf{r}_1 - \mathbf{r}_2)^2} + \mathrm{H.c.} \right] \tag{13}$$

In terms of this quantity, Eq. (12) writes

$$\int \mathcal{D}[\theta, \phi] \; e^{-S_0(\ell)} \left[1 + I(\ell) + \ldots\right] = \int \mathcal{D}[\theta, \phi] \; e^{-S_0(\ell + d\ell)} \left[1 + I(\ell + d\ell) + \ldots\right] \tag{14}$$

Note that the rescaling $a(\ell) \rightarrow a(\ell + d\ell)$ changes the lower integration limit in Eq. (13), and we can split the integrals as

$$\int_{a(\ell+d\ell)<|\mathbf{r}_1-\mathbf{r}_2|} = \int_{a(\ell)<|\mathbf{r}_1-\mathbf{r}_2|} - \int_{a(\ell)<|\mathbf{r}_1-\mathbf{r}_2|<a(\ell+d\ell)} . \tag{15}$$

This allows to split the 2nd-order contribution into

$$I\left(\ell+d\ell\right)=I_1\left(\ell+d\ell\right)-I_2\left(\ell+d\ell\right),$$

where $I_1\left(\ell+d\ell\right)$ is identical to Eq. (13), provided we adjust the prefactors as

$$\frac{\tilde{\Delta}\left(\ell+d\ell\right)}{\left(a\left(\ell+d\ell\right)\right)^{2-1/K}}=\frac{\tilde{\Delta}\left(\ell\right)}{\left(a\left(\ell\right)\right)^{2-1/K}},\tag{16}$$

$$\frac{\tilde{\alpha}\left(\ell+d\ell\right)}{\left(a\left(\ell+d\ell\right)\right)^{1-2K}}=\frac{\tilde{\alpha}\left(\ell\right)}{\left(a\left(\ell\right)\right)^{1-2K}},\tag{17}$$

From here, the RG-flow Eqs. (8) in the main text:

$$\frac{d\tilde{\Delta}\left(\ell\right)}{d\ell}=\left(2-\frac{1}{K\left(\ell\right)}\right)\tilde{\Delta}\left(\ell\right),\tag{18}$$

$$\frac{d\tilde{\alpha}\left(\ell\right)}{d\ell}=\left(1-2K\left(\ell\right)\right)\tilde{\alpha}\left(\ell\right),\tag{19}$$

are obtained.

On the other hand, the term $I_2\left(\ell+d\ell\right)$ is the integral evaluated near the boundary, i.e.,

$$\begin{aligned}I_2\left(\ell+d\ell\right)=&\frac{1}{2}\frac{\left(\tilde{\Delta}\left(\ell\right)\right)^2}{\left(a\left(\ell\right)\right)^{4-2/K}}\int_{a(\ell)<|\mathbf{r}_1-\mathbf{r}_2|<a(\ell+d\ell)}d^2\mathbf{r}_1d^2\mathbf{r}_2\,\left(\frac{2\pi}{L}\right)^{2/K}\left[:e^{i2\tilde{\theta}(\mathbf{r}_1)/\sqrt{K}}::e^{-i2\tilde{\theta}(\mathbf{r}_2)/\sqrt{K}}:+\text{H.c.}\right]\\&+\frac{1}{2}\frac{\tilde{\alpha}\left(\ell\right)}{\left(a\left(\ell\right)\right)^{1-2K}}\int_{a(\ell)<|\mathbf{r}_1-\mathbf{r}_2|<a(\ell+d\ell)}d^2\mathbf{r}_1d^2\mathbf{r}_2\,\delta\left(x_1-x_2\right)\left(\frac{2\pi}{L}\right)^{2K}\left[\frac{:e^{i2\sqrt{K}\tilde{\phi}(\mathbf{r}_1)}::e^{-i2\sqrt{K}\tilde{\phi}(\mathbf{r}_2)}:}{\left(\mathbf{r}_1-\mathbf{r}_2\right)^2}+\text{H.c.}\right]\end{aligned}\tag{20}$$

and is easy to see that this term renormalizes the fixed-point action $S_0\left(\ell+d\ell\right)$, as can be seen reexponenting this term in the expression of the partition function. We obtain

$$\begin{aligned}\int\mathcal{D}\left[\theta,\phi\right]\,e^{-S_0(\ell)}\left[1+I\left(\ell\right)+\ldots\right]&\approx\int\mathcal{D}\left[\theta,\phi\right]\,e^{-S_0(\ell+d\ell)}\left[1+I_1\left(\ell+d\ell\right)-I_2\left(\ell+d\ell\right)+\ldots\right],\\&=\int\mathcal{D}\left[\theta,\phi\right]\,e^{-S_0(\ell+d\ell)-I_2(\ell+d\ell)}\left[1+I_1\left(\ell+d\ell\right)+\ldots\right].\end{aligned}$$

Using the previous result $I\left(\ell\right)=I_1\left(\ell+d\ell\right)$ upon rescaling of the parameters as in Eqs. (16) and (17), note that in order to preserve the partition function, we must impose:

$$S_0\left(\ell\right)=S_0\left(\ell+d\ell\right)+I_2\left(\ell+d\ell\right).\tag{21}$$

Next, we perform an operator product expansion (OPE) in Eq. (20). To that end, it is convenient to introduce relative and center-of-mass coordinates,

$$\begin{aligned}\mathbf{r}&=\mathbf{r}_1-\mathbf{r}_2,\\\mathbf{R}&=\frac{1}{2}\left(\mathbf{r}_1+\mathbf{r}_2\right).\end{aligned}$$

In the limit $\mathbf{r} \to 0$, the product of vertex operators appearing in (13) can be expanded as:

$$
\begin{aligned}
: e^{i2\sqrt{K}\tilde{\phi}\left(\mathbf{R}+\frac{\mathbf{r}}{2}\right)} :: e^{-i2\sqrt{K}\tilde{\phi}\left(\mathbf{R}+\frac{\mathbf{r}}{2}\right)} : &= \left(\frac{L}{2\pi}\right)^{2K} \frac{: e^{i2\sqrt{K}\tilde{\phi}\left(\mathbf{R}+\frac{\mathbf{r}}{2}\right)} e^{-i2\sqrt{K}\tilde{\phi}\left(\mathbf{R}+\frac{\mathbf{r}}{2}\right)} :}{\left(x^2 + v^2\left(\tau+a\right)^2\right)^K} \\
&= \left(\frac{L}{2\pi}\right)^{2K} \frac{: e^{i2\sqrt{K}\left[\tilde{\phi}\left(\mathbf{R}+\frac{\mathbf{r}}{2}\right)-\tilde{\phi}\left(\mathbf{R}-\frac{\mathbf{r}}{2}\right)\right]} :}{\left(x^2 + v^2\left(\tau+a\right)^2\right)^K} \\
&\xrightarrow[\mathbf{r}\to 0]{} \left(\frac{L}{2\pi}\right)^{2K} \frac{: \sum_{n=0}^{\infty} \frac{\left(i2\sqrt{K}\right)^n}{n!} \left[\partial_x \tilde{\phi}\left(\mathbf{R}\right) x + \partial_\tau \tilde{\phi}\left(\mathbf{R}\right)\tau\right]^n + \cdots :}{\left(x^2 + v^2\left(\tau+a\right)^2\right)^K}
\end{aligned}
\tag{22}
$$

$$
\begin{aligned}
: e^{i2\tilde{\theta}\left(\mathbf{R}+\frac{\mathbf{r}}{2}\right)/\sqrt{K}} :: e^{-i2\tilde{\theta}\left(\mathbf{R}-\frac{\mathbf{r}}{2}\right)/\sqrt{K}} : &= \left(\frac{L}{2\pi}\right)^{2/K} \frac{: e^{i2\tilde{\theta}\left(\mathbf{R}+\frac{\mathbf{r}}{2}\right)/\sqrt{K}} e^{-i2\tilde{\theta}\left(\mathbf{R}-\frac{\mathbf{r}}{2}\right)/\sqrt{K}} :}{\left(x^2 + v^2\left(\tau+a\right)^2\right)^{1/K}} \\
&= \left(\frac{L}{2\pi}\right)^{2/K} \frac{: e^{i2\left[\tilde{\theta}\left(\mathbf{R}+\frac{\mathbf{r}}{2}\right)-\tilde{\theta}\left(\mathbf{R}-\frac{\mathbf{r}}{2}\right)\right]/\sqrt{K}} :}{\left(x^2 + v^2\left(\tau+a\right)^2\right)^{1/K}} \\
&= \left(\frac{L}{2\pi}\right)^{2/K} \frac{: \sum_{n=0}^{\infty} \left(\frac{2i}{\sqrt{K}}\right)^n \left[\partial_x \tilde{\theta}\left(\mathbf{R}\right) x + \partial_\tau \tilde{\theta}\left(\mathbf{R}\right)\tau\right]^n + \cdots :}{\left(x^2 + v^2\left(\tau+a\right)^2\right)^{1/K}}
\end{aligned}
\tag{23}
$$

With these results, the integral (20) becomes

$$
\begin{aligned}
I_2\left(\ell+d\ell\right) &= \frac{\left(\tilde{\Delta}\left(\ell\right)\right)^2}{\left(a\left(\ell\right)\right)^{4-2/K}} \int d\mathbf{R} \int_{a(\ell)<|\mathbf{r}_1-\mathbf{r}_2|<a(\ell+d\ell)} d^2\mathbf{r}\, \frac{: 1 + \frac{1}{2!}\left(i\frac{2}{\sqrt{K}}\right)^2 \left[\left(\partial_x \tilde{\theta}\left(\mathbf{R}\right)\right)^2 x^2 + \left(-iv\partial_x \tilde{\phi}\left(\mathbf{R}\right)\right)^2 \tau^2\right] + \cdots :}{\left(x^2 + v^2\left(\tau+a\right)^2\right)^{1/K}} \\
&+ \frac{\tilde{\alpha}\left(\ell\right)}{\left(a\left(\ell\right)\right)^{1-2K}} \int d\mathbf{R} \int_{a(\ell)<|\mathbf{r}_1-\mathbf{r}_2|<a(\ell+d\ell)} d^2\mathbf{r}\, \frac{\delta\left(x\right)}{\mathbf{r}^2} \frac{: 1 + \frac{\left(i2\sqrt{K}\right)^2}{2!} \left[\partial_x \tilde{\phi}\left(\mathbf{R}\right) x + \partial_\tau \tilde{\phi}\left(\mathbf{R}\right)\tau\right]^2 + \cdots :}{\left(x^2 + v^2\left(\tau+a\right)^2\right)^K} \\
&\approx \frac{\left(\tilde{\Delta}\left(\ell\right)\right)^2}{\left(a\left(\ell\right)\right)^{4-2/K}} \int d\mathbf{R} \int_0^{2\pi} d\varphi \int_{a(\ell)}^{a(\ell+d\ell)} dr\, r \frac{: 1 + \frac{1}{2!}\left(i\frac{2}{\sqrt{K}}\right)^2 r^2 \left[\cos^2\varphi \left(\partial_x \tilde{\theta}\left(\mathbf{R}\right)\right)^2 - \sin^2\varphi \left(\partial_x \tilde{\phi}\left(\mathbf{R}\right)\right)^2\right] + \cdots :}{\left(r^2\right)^{1/K}} \\
&+ \frac{\tilde{\alpha}\left(\ell\right)}{\left(a\left(\ell\right)\right)^{1-2K}} \int d\mathbf{R} \int_{a(\ell)}^{a(\ell+d\ell)} d\left(v\tau\right) \left[\frac{: 1 + \frac{\left(i2\sqrt{K}\right)^2}{2!} \left[-v^2\tau^2 \left(\partial_x \tilde{\theta}\left(\mathbf{R}\right)\right)^2\right] + \ldots}{\left(v\tau\right)^{2+2K}}\right] \\
&= -\frac{2\pi}{K\left(\ell\right)} \left(\tilde{\Delta}\left(\ell\right)\right)^2 d\ell \int d\mathbf{R} \left\{: \left[\left(\partial_x \tilde{\theta}\left(\mathbf{R}\right)\right)^2 - \left(\partial_x \tilde{\phi}\left(\mathbf{R}\right)\right)^2\right] + \cdots :\right\} \\
&+ 2K\left(\ell\right) \tilde{\alpha}\left(\ell\right) d\ell \int d\mathbf{R} \left[: \left(\partial_x \tilde{\theta}\left(\mathbf{R}\right)\right)^2 + \ldots\right] + \text{cst.},
\end{aligned}
$$

where we have used the equation of motion for the bosonic fields

$$
\begin{aligned}
\partial_\tau \tilde{\phi}\left(x,\tau\right) &= \left[H, \tilde{\phi}\left(x,\tau\right)\right] = -iv\partial_x \tilde{\theta}\left(x,\tau\right), \\
\partial_\tau \tilde{\theta}\left(x,\tau\right) &= \left[H, \tilde{\theta}\left(x,\tau\right)\right] = -iv\partial_x \tilde{\phi}\left(x,\tau\right).
\end{aligned}
$$

We can now return to Eq. (21), and using the expression of the original fields Eqs. (6) and (7), we can equate the coefficients of $\left(\partial_x \phi\right)^2$ and $\left(\partial_x \theta\right)^2$ appearing on both sides of (21). |We obtain respectively

$$\frac{v(\ell)}{2\pi K(\ell)} = \frac{v(\ell+d\ell)}{2\pi K(\ell+d\ell)} + \frac{2\pi}{K^2(\ell)}\left(\tilde{\Delta}(\ell)\right)^2 v(\ell)\,d\ell,$$
$$\frac{v(\ell)K(\ell)}{2\pi} = \frac{v(\ell+d\ell)K(\ell+d\ell)}{2\pi} + \left(-\frac{2\pi}{K(\ell)}\left(\tilde{\Delta}(\ell)\right)^2 v(\ell)\,d\ell + 2K(\ell)\,\tilde{\alpha}(\ell)\,v(\ell)\,d\ell\right)K(\ell).$$

From here we derive the set of differential equations

$$\frac{d\left(\frac{v(\ell)}{K(\ell)}\right)}{d\ell} = \frac{1}{K(\ell)}\frac{dv(\ell)}{d\ell} - \frac{v(\ell)}{K^2(\ell)}\frac{dK(\ell)}{d\ell} = -\frac{(2\pi)^2}{K^2(\ell)}v(\ell)\left(\tilde{\Delta}(\ell)\right)^2,$$
$$\frac{d(v(\ell)K(\ell))}{d\ell} = K(\ell)\frac{dv(\ell)}{d\ell} + v(\ell)\frac{dK(\ell)}{d\ell} = +(2\pi)^2 v(\ell)\left(\tilde{\Delta}(\ell)\right)^2 - 4\pi K^2(\ell)\,v(\ell)\,\tilde{\alpha}(\ell),$$

and from here,

$$K(\ell)\frac{dv(\ell)}{d\ell} - v(\ell)\frac{dK(\ell)}{d\ell} = -(2\pi)^2 v(\ell)\left(\tilde{\Delta}(\ell)\right)^2,$$
$$K(\ell)\frac{dv(\ell)}{d\ell} + v(\ell)\frac{dK(\ell)}{d\ell} = +(2\pi)^2 v(\ell)\left(\tilde{\Delta}(\ell)\right)^2 - 4\pi K^2(\ell)\,v(\ell)\,\tilde{\alpha}(\ell).$$

Solving for $\frac{dv(\ell)}{d\ell}$ and $\frac{dK(\ell)}{d\ell}$, we finally obtain

$$\frac{dv(\ell)}{d\ell} = -2\pi K(\ell)\,v(\ell)\,\tilde{\alpha}(\ell), \tag{24}$$
$$\frac{dK(\ell)}{d\ell} = (2\pi)^2\left(\tilde{\Delta}(\ell)\right)^2 - 2\pi K^2(\ell)\,\tilde{\alpha}(\ell). \tag{25}$$

III. PERTURBATION THEORY IN THE STRONGLY DISSIPATIVE LIMIT

In this section, we derive the effective Hamiltonian for a two-site toy model in the strongly dissipative limit based on the perturbation theory. The Hamiltonian of the two-site system can be expressed (in terms of the spin language) as:

$$H = H_s + \sum_{i=1,2}\sum_k [c_k\sigma_i^z X_{ik} + \frac{P_{ik}^2}{2m_k} + \frac{1}{2}m_k\omega_k^2 X_{ik}^2] \tag{26}$$

where $H_s = -J\sigma_1^x\sigma_2^x - \frac{\mu}{2}[\sigma_1^z + \sigma_2^z]$ is the system Hamiltonian. In the limit $J = \mu = 0$, the two sites are decoupled. For each site, the ground state are two-fold degenerate, denoted as "dressed" spin states: $|\tilde{\uparrow}\rangle = |\uparrow\rangle \otimes_{k=1}^{k_c} |\Psi_k^+\rangle$ and $|\tilde{\downarrow}\rangle = |\downarrow\rangle \otimes_{k=1}^{k_c} |\Psi_k^-\rangle$, where $|\uparrow/\downarrow\rangle$ is the eigenstate of the system spin σ_z, $|\Psi_k^\pm\rangle$ is the bosonic coherent state of the kth-mode harmonic oscillator: $|\Psi_k^\pm\rangle = \frac{1}{\pi^{\frac{1}{4}}\sqrt{l_k}}\exp[-\frac{(x\pm\xi_k)^2}{2l_k^2}]$ with $l_k = \sqrt{\frac{1}{m_k\omega_k}}$ and $\xi_k = \frac{c_k}{2m_k\omega_k^2}$.
In the strong dissipative limit where $J, \mu \ll c_k, \omega_k$, H_s can be treated as a perturbation. By performing the standard perturbation analysis, one can derive the effective Hamiltonian in the unperturbed basis $|\tilde{\uparrow}_1\tilde{\uparrow}_2\rangle$, $|\tilde{\uparrow}_1\tilde{\downarrow}_2\rangle$,$|\tilde{\downarrow}_1\tilde{\uparrow}_2\rangle$, $|\tilde{\downarrow}_1\tilde{\downarrow}_2\rangle$:

$$\tilde{H} = -\tilde{J}\tilde{\sigma}_1^x\tilde{\sigma}_2^x - \frac{\tilde{\mu}}{2}[\tilde{\sigma}_1^z + \tilde{\sigma}_2^z] \tag{27}$$

where the Pauli operators $\tilde{\sigma}^{x,y,z}$ operate in the Hilbert space spanned by the "dressed" spin basis $|\tilde{\uparrow}\rangle$ and $|\tilde{\downarrow}\rangle$, and the diagonal matrix elements are not normalized in the first order perturbation $\tilde{\mu} = \mu$, while the off-diagonal ones:

$$\tilde{J} = J\prod_k \langle\Psi_{1,k}^+|\Psi_{1,k}^-\rangle\langle\Psi_{2,k}^+|\Psi_{2,k}^-\rangle = J\prod_k e^{-\frac{2\xi_k^2}{l_k^2}} = Je^{-\sum_k \frac{c_k^2}{m_k\omega_k^3}} = Je^{-\int\frac{J(\omega)}{\omega^2}} = J(\frac{a}{\Lambda})^\alpha \tag{28}$$

where $J(\omega) = \sum_k \delta(\omega - \omega_k)\frac{c_k^2}{m_k\omega_k} = \alpha\omega$ for $a < \omega < \Lambda$ with $\Lambda(a)$ the ultraviolet (infrared) frequency cut-off. From Eq. (28) we can find that in the strong dissipative limit, the off diagonal coupling are strongly suppressed by dissipation.
